# Clinical Features and Outcomes of Monobacterial and Polybacterial Episodes of Ventilator-Associated Pneumonia Due to Multidrug-Resistant *Acinetobacter baumannii*

**DOI:** 10.3390/antibiotics11070892

**Published:** 2022-07-04

**Authors:** Dalia Adukauskiene, Ausra Ciginskiene, Agne Adukauskaite, Despoina Koulenti, Jordi Rello

**Affiliations:** 1 Medical Academy, Lithuanian University of Health Sciences, 44307 Kaunas, Lithuania; daliaadu@gmail.com; 2 Department of Cardiology and Angiology, University Hospital of Innsbruck, 6020 Innsbruck, Austria; agne.adukauskaite@tirol-kliniken.at; 3 Second Critical Care Department, Attikon University Hospital, 12462 Athens, Greece; deskogr@yahoo.gr; 4 UQ Centre for Clinical Research (UQCCR), Faculty of Medicine, The Univesrity of Queensland, Brisbane 4029, Australia; 5 Vall d‘Hebron Institute of Research, Vall d‘Hebron Campus Hospital, 08035 Barcelona, Spain; jrello@crips.es; 6 Clinical Research, CHU Nîmes, 30900 Nîmes, France

**Keywords:** *Acinetobacter baumannii*, antibiotic optimisation, antibiotic stewardship (AMS), aspiration pneumonia, colistin, hospital-acquired pneumonia (HAP), multidrug-resistance (MDR), mortality, non-fermentative Gram-negative bacilli (GNB), polymicrobial, pneumonia resolution, ventilator-associated pneumonia (VAP)

## Abstract

Multidrug-resistant *A. baumannii* (MDRAB) VAP has high morbidity and mortality, and the rates are constantly increasing globally. Mono- and polybacterial MDRAB VAP might differ, including outcomes. We conducted a single-center, retrospective (January 2014–December 2016) study in the four ICUs (12–18–24 beds each) of a reference Lithuanian university hospital, aiming to compare the clinical features and the 30-day mortality of monobacterial and polybacterial MDRAB VAP episodes. A total of 156 MDRAB VAP episodes were analyzed: 105 (67.5%) were monomicrobial. The 30-day mortality was higher (*p* < 0.05) in monobacterial episodes: overall (57.1 vs. 37.3%), subgroup with appropriate antibiotic therapy (50.7 vs. 23.5%), and subgroup of XDR *A. baumannii* (57.3 vs. 36.4%). Monobacterial MDRAB VAP was associated (*p* < 0.05) with Charlson comorbidity index ≥3 (67.6 vs. 47.1%), respiratory comorbidities (19.0 vs. 5.9%), obesity (27.6 vs. 9.8%), prior hospitalization (58.1 vs. 31.4%), prior antibiotic therapy (99.0 vs. 92.2%), sepsis (88.6 vs. 76.5%), septic shock (51.9 vs. 34.6%), severe hypoxemia (23.8 vs. 7.8%), higher leukocyte count on VAP onset (median [IQR] 11.6 [8.4–16.6] vs. 10.9 [7.3–13.4]), and RRT need during ICU stay (37.1 vs. 17.6%). Patients with polybacterial VAP had a higher frequency of decreased level of consciousness (*p* < 0.05) on ICU admission (29.4 vs. 14.3%) and on VAP onset (29.4 vs. 11.4%). We concluded that monobacterial MDRAB VAP had different demographic/clinical characteristics compared to polybacterial and carried worse outcomes. These important findings need to be validated in a larger, prospective study, and the management implications to be further investigated.

## 1. Introduction

Ventilator-associated pneumonia (VAP) is the most frequent infection in the intensive care unit (ICU) with a significant impact on the morbidity and mortality of critically ill patients, as VAP development has been associated with increased duration of mechanical ventilation (MV), prolonged ICU and hospital stay, increased consumption of antibiotics, and increased health-care costs [1,2]. The reported incidence varies significantly in the relevant literature, from 1–2.5 cases per 1000 ventilator-days in the USA to 116 cases per 1000 ventilator-days in the Southeast Asian Region, and this variation might be, at least partially, attributed to differences in prevention measures, definitions used, and case mix [3,4]. It has been demonstrated that the clinical manifestation and outcomes of VAP might vary depending on the pathogen. There are multiple VAP studies on specific pathogens describing their clinical, management, and outcome-related aspects without, however, considering the mono- or polybacterial VAP origin [5,6].

The clinical importance of *Acinetobacter baumannii* (*A. baumannii*) has been steadily increasing on a worldwide level. It has established a niche in the hospital environment causing a variety of severe infections, especially in the critical care setting. It is usually a difficult-to-treat pathogen displaying a high resistance profile and has been associated with high mortality, morbidity, and health care costs [5,6,7,8,9,10,11,12,13,14,15,16,17,18,19,20,21,22]. Although some controversy exists in the relevant literature, it seems that the high mortality of infections caused by multidrug-resistant (MDR) pathogens may be related not only to the bacterial resistance but also to the severity of illness and the appropriateness and timeliness of antibacterial treatment [1,5,8,9,10,11,12].

Regarding VAP, *A. baumannii* is one of the most common pathogens of both mono- and polybacterial VAP [6,7]. The association between mono- and polybacterial VAP caused by MDRAB and patients’ mortality has not been thoroughly investigated yet. We believe that it would be methodologically more accurate to analyze the monobacterial VAP cases separately from the polybacterial ones in order to reliably estimate the association between a pathogen and the clinical presentation and outcomes of VAP. Our hypothesis was that the mortality and clinical characteristics of patients with VAP due to MDRAB differ between mono- and polybacterial cases. Hence, the primary objective of our study was to compare the 30-day mortality between patients with mono- and polybacterial MDRAB VAP, while the secondary objective was to compare their clinical features.

## 2. Methods and Materials

A retrospective cohort study was conducted in the four adult ICUs (medical-surgical, neurosurgical, cardiosurgical, and coronary care; 12–18–24 beds each) of the Hospital of Lithuanian University of Health Sciences Kaunas Clinics, a reference hospital that is the largest of the country (2300 beds). The study was approved by Kaunas Regional Biomedical Research Ethics Committee (No BEC-MF-156 and No P1-BE-2-13/2016). The need for written consent was waived due to the observational nature of the study.

The medical records of all patients admitted to the ICUs over a three-year period (from January 2014 to December 2016) were reviewed. Inclusion criteria were as follows: (1) age ≥ 18 years and (2) the first episode of VAP due to MDRAB. Exclusion criteria were: (1) polybacterial cases with Gram-positive co-pathogens, (2) neutropenia, and (3) deceased within the first 24 h after VAP onset.

Pneumonia was considered to be ventilator-associated when it occurred 48 h or more after intubation and onset of mechanical ventilation. Clinical diagnosis of VAP was made according to 2005 American Thoracic Society/Infectious Diseases Society of America (ATS/IDSA) criteria [23]. Sepsis status was diagnosed according to Sepsis-2 criteria [24]. The severity of illness was assessed on ICU admission and on VAP diagnosis using the Sequential Organ Failure Assessment (SOFA) and Simplified Acute Physiology Score II (SAPS II) scores. The identification of *A. baumannii* isolates and antibiotic susceptibility was performed according to the European Committee on Antimicrobial Susceptibility Testing (EUCAST) guidelines [25]. *A. baumannii* isolates were defined as MDR and extensively drug-resistant (XDR) according to an international expert proposal for the interim standard definitions for acquired resistance criteria, i.e., MDR when they were non-susceptible to at least one agent in three or more antimicrobial categories, and XDR when they were non-susceptible to at least one agent in all but two or fewer antimicrobial categories [26]. XDR isolates represent a sub-group of MDRs [26].

Demographics, clinical and laboratory data for each VAP case were recorded, including: (1) data of the first MDRAB positive tracheal aspirate culture, bacterial load (moderate/heavy growth), and drug resistance of *A. baumannii* strains; (2) age, gender, type of admission (medical/surgical), and comorbidities; (3) red blood cell (RBC) transfusion, reintubation, tracheostomy, and the need for renal replacement therapy (RRT) during the ICU stay; (4) sepsis, septic shock, oxygenation, temperature, inflammatory, and acid-base status on VAP diagnosis; (5) severity of illness on ICU admission and on VAP onset; (6) the use of intravenous (IV) antibiotics within the prior 90 days; (7) outcome: discharge or death at day 30 after VAP onset. The SOFA score was used to define organ dysfunction (>0) and organ failure (>2) both on ICU admission and on VAP onset. The baseline comorbidities were assessed using the Charlson comorbidity index (CCI), and the sepsis status using SEPSIS 2 criteria. Admission was considered as surgical in patients who had undergone surgery in the preceding four weeks. Antibacterial therapy was considered appropriate when at least one antibacterial agent, to which all causative pathogens were susceptible in vitro, was administered.

The mortality was defined as all-cause mortality within 30-day period after VAP diagnosis. To rule out the potential impact of several factors on mortality, the patients were grouped based on their disease severity on VAP diagnosis (SOFA < 8 vs. SOFA > 7), antimicrobial resistance of *A. baumannii* strains (MDR vs. XDR), appropriateness (appropriate vs. inappropriate) and timeliness (less vs. more than 48 h from VAP onset) of antibacterial treatment.

### Statistical Analysis

The variables were summarized as frequencies and percentages or medians and interquartile range (IQR). Mann–Whitney non-parametric test, Pearson’s chi-square test, or two-tailed Fisher’s exact test were performed to detect the differences between groups as appropriate. Mortality was analyzed both, as a binary outcome (survivor/non-survivor) and as survival time data. In the survival analysis, Kaplan–Meier estimates of the probability of survival were obtained, and survival curves were compared between groups using the Log Rank test. Two-sided *p* values of <0.01 and <0.05 were considered statistically significant for the Log Rank test and all other analyses, respectively. Statistical analysis was performed using the Statistical Package for the Social Sciences (SPSS), version 24 (SPSS, Chicago, IL, USA).

## 3. Results

A total of 156 VAP cases due to MDRAB were included in the analysis: 105 (67.3%) monobacterial and 51 (32.7%) polybacterial episodes, *p* < 0.001. In association with *A. baumannii*, one co-pathogen was found in 40 (25.6%), and two co-pathogens in 11 (7.1%) cases of polybacterial VAP; *Klebsiella* spp. and *P. aeruginosa* were the most frequently isolated co-pathogens. Most of *A. baumannii* strains (85.3%) were found to be of XDR profile (*p* < 0.001). All of them were susceptible to colistin, however, the vast majority (>90%) were resistant to piperacillin/tazobactam, cephalosporins, and carbapenems.

Patients with monobacterial episodes had more frequently prior antibiotic use, particularly carbapenems and antifungals, higher CCI, higher white blood cells count on VAP onset, and more frequent RRT during the ICU stay. Moreover, they had a higher respiratory SOFA score on ICU admission and more severe hypoxia on VAP onset, as depicted by the PaO_2_/FiO_2_ ratio (detailed comparison in Table 1).

Patients with monobacterial VAP due to MDRAB had higher mortality compared to those with polybacterial VAP, even after controlling for factors that may affect mortality. The detailed characteristics of 30-day mortality are provided in Table 2.

**Figure 1:**
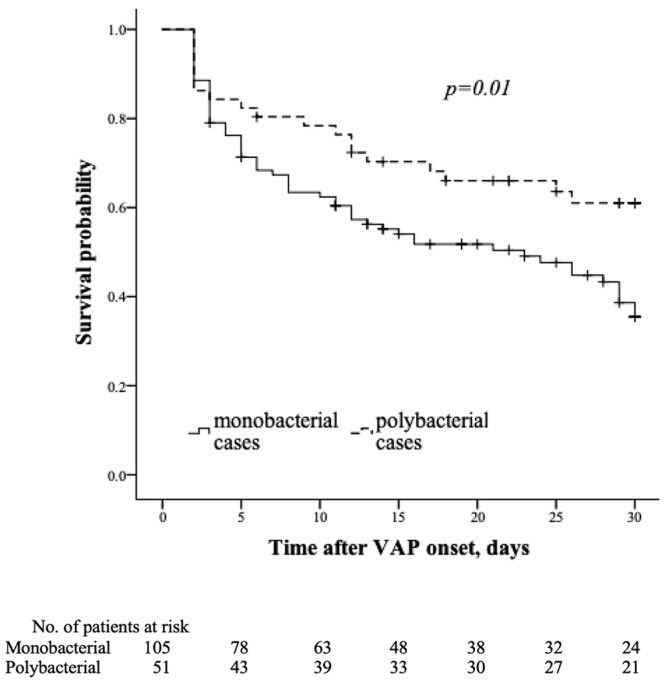
Kaplan–Meier survival curves for time to death in monobacterial vs. polybacterial VAP due to MDR *A. baumannii* (censored at 30 days).

The time to death (censored at day 30) was also shorter in the group with monobacterial VAP due to MDRAB, *p* = 0.01 (Figure 1).

## 4. Discussion

The key new finding of this analysis: the 30-day mortality rate in VAP due to MDRAB was higher in monobacterial compared to polybacterial cases. Mortality remained higher or showed the trend to be higher even after adjusting for the impact of disease severity, adequacy of treatment, timeliness of treatment, and the resistance profile of *A. baumannii* strains. Moreover, mono- and polybacterial cases of VAP had different demographic and clinical characteristics.

Previous studies show high all-cause mortality due to MDRAB VAP, however, it remains unclear whether, and to what degree, the poor outcomes are associated with the case mix, the underlying comorbidities, the multi-organ dysfunction, the pathogenicity and antibiotic resistance of *A. baumannii* strains, or other factors, such as the presence of co-pathogens in case of polybacterial infection [27,28,29,30]. The role of a specific pathogen in the disease course and outcome of polybacterial infections is difficult to be estimated due to possible positive or negative bacterial interactions. Although MDRAB is becoming one of the most common pathogens of VAP in several countries, so far, we have found no study comparing the impact of monobacterial and polybacterial origin of VAP on patient mortality. The current study adds knowledge to the field, demonstrating that the 30-day mortality was statistically significantly higher (57.1% vs. 37.3%) in the group of monobacterial VAP compared to polybacterial cases. Similarly, in the study of Brewer et al. [31] that compared mono- and polybacterial *P. aeruginosa* VAP, there was a trend for higher mortality in monobacterial cases (78.0% vs. 53.0%, *p* = 0.15). In contrast, Combes et al. [32] did not identify any differences in 30-day mortality between mono- and polybacterial VAP cases. Nevertheless, it is difficult to compare these results with ours, as they did not analyze MDRAB cases exclusively, but VAP caused by various pathogens, including Gram-positive ones, without specifying their antimicrobial susceptibility. Furthermore, in the study of Combes et al., *Acinetobacter* species accounted for less than 6% of all pathogens [32].

To assess the possible impact of confounders affecting mortality, we stratified monobacterial and polybacterial VAP cases into subgroups according to the severity of illness, appropriateness, and timeliness of antibacterial treatment, and MDR profile (MDR vs. XDR). When we controlled for the severity of illness (SOFA < 8 vs. SOFA > 7), we noted a trend for increased mortality in both sub-groups of monobacterial VAP cases. Both recent studies of Chang et al. [11] on HAP/VAP, with MDRAB as the most common pathogen, and of the ID-IRI group [9] on *A. baumannii* VAP demonstrated an association between reduced mortality and appropriate treatment. Nonetheless, these studies did not analyze whether this association persisted after assessing pneumonia’s mono- vs. polybacterial origin. In our analysis, 30-day mortality was found to be significantly higher in the monobacterial cases, even in the cases with appropriate antibacterial treatment. When comparing the additional impact of both disease severity and appropriateness of treatment, we revealed higher 30-day mortality in monobacterial VAP cases in the sub-group with the more severe disease, while a trend for higher mortality was shown in the lower disease severity sub-group, too (*p* = 0.07). Early appropriate therapy was not associated with mortality differences in mono- vs. polybacterial VAP cases. On the other hand, in delayed appropriate treatment, a trend for increased 30-day mortality in monobacterial VAP cases was identified.

The fact that the monobacterial MDRAB VAP had a worse outcome compared to the polybacterial cases might be explained by possible higher pathogen virulence and, consequently, a worse disease course. There is evidence that the same pathogen in a polybacterial environment might become less virulent compared to a monobacterial setting due to pathogen competition in the process of infection [33,34,35]. Bacteria can form dynamic polymicrobial communities with complex interactions, either co-operative or competitive [33,34,35]. Competition between bacteria may be expressed in several ways, including consuming resources to limit the growth of the competitor and even production of intrinsic antimicrobial compounds [33,34,35,36]. If *A. baumannii* is the only causative agent of VAP, there is no need for an intraspecies fight, and authentic virulence is revealed. Furthermore, there is evidence in the literature that the co-existence of several pathogens may influence their physiological functions, including their susceptibility to specific antimicrobial agents [33]. Further research is needed to address pending questions regarding the molecular mechanisms behind the interactions between co-existing pathogens in terms of virulence and antimicrobial susceptibility and between co-existing pathogens and host immune responses [35].

Although it has been speculated that bacteria lose their fitness and virulence by gaining antibiotic resistance, several studies that report very high infection-related mortality contrast this speculation [30,36,37,38]. Almomani et al. [38] had reported mortality of 42.0% in MDRAB VAP, while the reported mortality of Choi et al. [30] in XDR *A. baumannii* VAP was 23.8%. The high mortality of monobacterial MDRAB VAP reported in our study corroborates the data that demonstrate the very aggressive nature of these bacteria.

A rapid spread of resistance of *A. baumannii* strains against most of the widely used antibiotics limits the therapeutic choices and make appropriate treatment challenging. Although a clear and unanimous consensus on MDRAB VAP treatment is still missing, the current ATS/IDSA hospital-acquired/ventilator-associated (HAP/VAP) guidelines [39] recommend reserving colistin for cases of *A. baumannii* sensitive only to this agent. Polymyxins effectively suppress *A. baumannii* growth in vitro, however, the following factors question their safety and efficacy in clinical use: narrow therapeutic window and only bacteriostatic effect, variable PK/PDs, side effects, and not clearly determined optimal dosage [40,41]. Moreover, strong evidence is missing on whether colistin should be used as monotherapy or in combination. It has been suggested that colistin, combined with other antibiotics, such as carbapenems, leads to better outcomes due to the synergism of the different antibiotic classes [42]. Many clinical studies on MDRAB VAP treatment with colistin or its combinations have been conducted, however, most of them were retrospective and heterogeneous (diverse patient populations, *A. baumannii* phenotypes and genotypes, and different antibiotic combinations), that is why their results cannot be easily compared, generalized, or translated into clinical practice. For instance, Tsioutis et al. [6] and Gu et al. [43] did not find any statistically significant differences in patient groups treated with colistin as monotherapy compared to colistin combinations with tigecycline, carbapenems, aminoglycosides, and trimethoprim/sulfamethoxazole. On the other hand, in the meta-analysis of Wang et al. [40], the mortality trend was shown to be higher in the colistin monotherapy group. A possible explanation for worse outcomes in the colistin monotherapy group could be the phenomenon that some subpopulations of MDRAB strains, which are resistant to colistin, are able to multiply in an environment with a much higher colistin concentration than the minimum inhibitory concentration (MIC) [43,44]. What also might contribute to worse outcomes is the poor colistin penetration into the lung tissue that leads to insufficient concentration in epithelial lining fluid when administered IV in the regular recommended dose. Therefore, it has been suggested to administer colistin, not only by IV, but also by inhalation [45]. It has been hypothesized that an aerosolized route of administration may contribute to a higher local colistin concentration and lower incidence of superinfections and side effects [46]. However, the meta-analyses of Florescu et al. [46] and Gu et al. [43] that compared the treatment efficacy of colistin administered alone IV vs. a combination of IV and inhaled colistin in VAP due to Gram-negative bacilli (GNB), did not reveal any significant differences in 28-day mortality or ICU- and hospital-related mortality, even after controlling for concomitant antibiotic treatment or the dose of IV colistin. Moreover, although the meta-analysis of 16 studies conducted by Valachis et al. [47] showed that a combination treatment of IV antibiotic plus inhaled colistin reduced the infection-related mortality (OR 0.58; 95% CI, 0.34–0.96), it did not show any significant influence on all-cause mortality (OR 0.74, 95% CI, 0.54–1.01). Nonetheless, this meta-analysis included observational cohorts only, and most studies were of low/very low quality of evidence with multiple risks of bias. Another systematic review and meta-analysis of randomized clinical trials restricted to VAP did not confirm the findings of Valachis et al. and, moreover, reported that aerosolized colistin might increase respiratory complications in severely hypoxemic patients [48]. A position paper of the European Society of Clinical Microbiology and Infectious Diseases (ESCMID) [49] recommended against the use of aerosolized antibiotics in addition to IV treatment as standard clinical practice. Among VAP cases caused by resistant pathogens, replacing systemic administration by aerosolization also failed to demonstrate further efficacy but showed reduced nephrotoxicity. In addition, the role of the aerosol device and standardization of administration is a major issue [50].

Treatment of infections due to MDR GNBs using colistin alternatives—sulbactam and tigecycline combinations—has been investigated [7,51,52]. However, most of the studies that compared the outcomes of *A. baumannii* infections treated with colistin vs. other antibiotics did not find any significant differences in mortality [32,35,40,43,45,46,47,53,54,55]. On the other hand, a meta-analysis of Jung et al. [7] on critically ill patients with pneumonia due to MDR/XDR *A. baumannii* investigated treatment efficacy with colistin compared to 15 other antibiotic regiments (sulbactam, high sulbactam dose, fosfomycin + IV colistin, high tigecycline dose, and IV + inhaled colistin) and found that a cefoperazone/sulbactam combination ranked higher than IV colistin for reducing all-cause mortality. In summary, these results should be interpreted cautiously, since the studies included not only VAP cases, but other infections, as well, and not all of them were *A. baumannii*-associated. Regarding cefoperazone/sulbactam, specifically, an open-label clinical trial in a patient with MDRAB HAP/VAP suggested that a combined pharmacokinetics/pharmacodynamics (PK/PD) index for both antibiotic agents [%(T > MIC_cpz_ × T > MIC_sul_)] was more appropriate for dose optimization than for a single agent PK/PD index [56]. Due to the limited therapeutic options, minocycline, alone or in combination, has also been used for MDRAB treatment, and a recent systematic review of MDRAB infections (the majority was pneumonia cases) has reported promising results that set the ground for further research [57]. New antibiotics are needed to reinforce the limited armamentarium against MDRAB [58].

The effect of the drug resistance profile of *A. baumannii* strains on mortality remains unclear. A large study by Lakbar et al. [10] that analyzed the association between antibiotic resistance and mortality in ICU-acquired pneumonia found that higher resistance of the causative pathogens increased the risk of death. On the contrary, Paramythiotou et al. [59] did not confirm the link between higher resistance of GNBs as VAP pathogens and increased mortality. The potential differences in the in vitro and in vivo activity of the antimicrobial agents might confound the association between drug resistance profiles and mortality. In our study, a higher resistance profile of *A. baumannii* strains, i.e., XDR, was significantly associated with increased mortality in monobacterial VAP cases. This relationship could be partly explained by the fact that the more resistant the pathogen, the less likely was the administration of appropriate antibacterial treatment.

Bringing novelty to the literature, we also compared the clinical characteristics of mono- and polybacterial MDRAB VAP on ICU admission and VAP onset. On ICU admission, the main findings were the more frequent multiple comorbidities (CCI ≥ 3, chronic respiratory disease, and obesity) and prior hospitalization in the monobacterial sub-group, whereas, on VAP onset, the patients with monobacterial MDRAB were in more severe conditions (e.g., leukocytosis, sepsis, septic shock, hypoxemia, and organ dysfunction). Moreover, the use of IV antibiotics, particularly carbapenems and antifungals, within 90-day before VAP onset was strongly associated with the monobacterial cases. The higher frequency of prior carbapenem use might have led to less diversity of bacterial flora in the lower respiratory tract and, thus, to monobacterial MDRAB VAP. The interaction between non-fermentative bacilli in the respiratory tract and yeast has been well documented, particularly for *P. aeruginosa*. Our findings support a close relationship between microbiome and microbiota diversity and the development of monobacterial episodes, with potentially important implications for antimicrobial stewardship. Ferrer et al. [27], in a methodologically quite similar study, also demonstrated that chronic underlying diseases were more prevalent among patients with monobacterial ICU-related pneumonia, but contrary to our study, it was found that hypoxemia and inflammatory response did not differ between mono- and polybacterial cases. However, a direct comparison of our study and the one of Ferrer et al. [27] would be inaccurate since the latter included ICU-related pneumonia cases with various pathogens/resistance profiles (not only Gram-negatives/MDRs). On the other hand, an interesting finding is that neurological impairment (as depicted by higher neurological SOFA) was significantly more frequent in the polybacterial VAP sub-group. Similar to our results, in the very recent study of Natarajan et al. [60], the decreased level of consciousness at the time of intubation (Glasgow Coma Scale < 8) was the single independent predictor of polybacterial VAP. The link between impaired consciousness and polybacterial VAP might be partly attributed to the fact that neurological dysfunction increases the risk of regurgitation and aspiration of polymicrobial-laden oropharyngeal secretion and gastric contents [60,61,62,63,64].

### Study Novelties and Limitations

To our knowledge, this is the first study to compare the mortality and the clinical characteristics of the mono- vs. the polybacterial episodes of VAP due to MDRAB. The results of our study indicate the important differences in the clinical characteristics and mortality of monobacterial vs. polybacterial MDRAB VAP. This information, along with the bacterial load (as depicted by quantitative or semi-quantitative cultures), could be taken into consideration when the results of respiratory cultures indicate polybacterial growth and we face the dilemma of whether *A. baumannii* represents a pathogen or an innocent by-stander (colonizer).

Our study also has some limitations. Although the data were collected in ICUs with a variety of case-mix in the country’s largest university hospital, it is still a single-center, retrospective study, so the results might not exactly depict the situation in other hospitals in the country, and further research is required before extrapolating them. On the other hand, regional hospitals use to transfer the critically ill patients to the tertiary care ICUs where the study was conducted, that is why we think our findings should represent well the whole country’s profile of VAP due to MDRAB. Furthermore, due to the quite limited size of the study cohort, some differences in the mortality of monobacterial in comparison with polybacterial episodes were found only in clinical relevance and did not reach statistical significance. Moreover, due to the limited sample size, neither sub-group analysis of MDRAB strains with different resistance profiles nor sub-group analysis of MDRAB co-infection with different pathogens could be performed. Finally, tracheobronchitis, VAP relapses, or superinfections were not recorded in our database, while the results of the respiratory cultures were not quantitative, facts that might have led to misclassification and under- or over-estimation of VAP.

## 5. Conclusions

Despite the limitation of being retrospective and single-centered, our study has provided important information on a field that the relevant literature is limited: whether monobacterial MDRAB VAP differs from the polybacterial one in clinical findings and mortality. Although the current study by itself, due to the limitations mentioned already, cannot inform change in practice, it can act as a platform for larger, well-designed, prospective studies that will further explore the difference between mono- and polymicrobial MDRAB VAP and their potential clinical implications, such as, whether—and to which subgroups of patients—the antimicrobial agent(s) could be withheld or discontinued when the results of the respiratory cultures depict polybacterial MDRAB VAP.

## Figures and Tables

**Table 1:** Characteristics of mono- and polybacterial cases of VAP due to MDRAB.

Variable	VAP Origin		
	Monobacterial n = 105	Polybacterial * n = 51	*p* Value
Age, years, median (IQR)	63 (54–72)	59 (52–67)	0.22
Sex, male, n (%)	61 (58.1)	32 (62.7)	0.61
Prior hospitalization within 90 days, n (%)	**61 (58.1)**	16 (31.4)	**<0.01**
Disease severity on ICU admission, median (IQR):			
▪ SOFA	7 (4–9)	7 (5–8)	0.64
▪ SAPS II	40.5 (33.0–56.0)	44.0 (35.0–54.0)	0.66
Admission to ICU from, n (%):			
▪ Community—ED	39 (37.1)	23 (45.1)	0.62
▪ Ward	37 (35.2)	15 (29.4)	
▪ Other ICU	29 (27.6)	13 (25.5)	
Duration of hospital stay prior to VAP onset, days, median (IQR)	13.0 (6.25–20.0)	11.0 (6.0–16.75)	1.00
Duration of ICU stay prior to VAP onset, days, median, IQR	8.5 (5.0–14.0)	9.0 (5.0- 13.75)	0.26
Admission, n (%):			
▪ Medical	66 (62.9)	30 (58.8)	0.73
▪ Surgical	39 (37.1)	21 (41.2)	
CCI ≥ 3, n (%)	**71 (67.6)**	24 (47.1)	**0.01**
Chronic illness, n (%):	86 (81.9)	39 (76.5)	0.43
▪ Cardiovascular	73 (69.5)	33 (64.7)	0.55
▪ Respiratory	**20 (19.0)**	3 (5.9)	**0.03**
▪ Neurological	8 (7.6)	2 (3.9)	0.50
▪ Renal	22 (21.0)	7 (13.7)	0.28
▪ Liver	9 (8.6)	4 (7.8)	0.89
▪ DM	18 (17.1)	7 (13.7)	0.59
▪ Oncology **	17 (16.2)	4 (7.8)	0.15
▪ Obesity ***	**29 (27.6)**	5 (9.8)	**0.01**
Organ failure on ICU admission, n (%):			
▪ SOFA respiratory ≥3	**75 (71.4)**	26 (50.0)	**0.01**
▪ SOFA cardiovascular ≥3	42 (40.0)	20 (39.2)	0.93
▪ SOFA neurologic ≥3	15 (14.3)	**15 (29.4)**	**0.03**
▪ SOFA renal ≥3	17 (16.2)	6 (11.8)	0.47
▪ SOFA liver ≥3	2 (1.9)	0 (0)	0.56
▪ SOFA coagulation ≥3	6 (5.7)	3 (5.9)	0.97
▪ MODS	53 (34)	22 (14.1)	0.39
Tracheostomy before VAP, n (%)	15 (14.3)	10 (19.6)	0.49
Reintubation before VAP, n (%)	13 (12.4)	4 (7.8)	0.39
RBC transfusion before VAP, n (%)	55 (51.9)	28 (57.1)	0.54
Use of IV antibiotics within 90 days, n (%):	**104 (99)**	47 (92.2)	**0.04**
▪ Penicillins	44 (41.9)	16 (31.4)	0.21
▪ Cephalosporins	86 (81.9)	39 (76.5)	0.43
▪ Fluoroquinolones	21 (20.0)	6 (11.8)	0.20
▪ Aminoglycosides	5 (4.8)	1 (2.0)	0.66
▪ Carbapenems	**9 (37.1)**	9 (17.6)	**0.02**
▪ Antifungal	**16 (15.2)**	1 (2.0)	**0.01**
Disease severity on VAP onset, median (IQR):			
▪ SAPS II score	45 (32.5–54.5)	43 (33.0–51.0)	0.40
▪ SOFA score	6 (4–10)	5 (4–8)	0.53
Organ failure on VAP onset, n (%):			
▪ SOFA respiratory ≥3	86 (81.9)	35 (68.6)	0.06
▪ SOFA cardiovascular ≥3	41 (39.0)	14 (27.5)	0.16
▪ SOFA neurological ≥3	12 (11.4)	**15 (29.4)**	**0.01**
▪ SOFA renal ≥3	18 (17.1)	6 (11.8)	0.38
▪ SOFA liver ≥3	4 (3.8)	0 (0)	0.30
▪ SOFA coagulation ≥3	9 (8.6)	2 (3.9)	0.29
▪ MODS	51 (48.6)	17 (33.3)	0.07
Sepsis on VAP onset, n (%)	**93 (88.6)**	39 (76.5)	**0.049**
Septic shock on VAP onset, n (%)	**54 (51.9)**	18 (34.6)	**0.041**
Temperature on VAP onset, n (%):			
▪ <36 °C	13 (12.4)	6 (11.8)	0.91
▪ ≥38.3 °C	48 (45.7)	22 (43.1)	0.76
Oxygenation index on VAP onset, n (%)			
▪ PaO_2_/FiO_2_ ≤ 300–>200	19 (18.1)	14 (27.5)	0.18
▪ PaO_2_/FiO_2_ ≤ 200–>100	62 (59.0)	34 (64.7)	0.36
▪ PaO_2_/FiO_2_ ≤ 100	**24 (22.9)**	3 (5.9)	**<0.01**
Inflammatory markers on VAP onset, median (IQR):			
▪ WBC, cells × 109/L	**12.2 (8.7–17.9)**	10.9 (7.3–13.4)	**0.03**
▪ CRP, mg/L	172 (113–241)	172 (119–235)	0.88
Acidosis on VAP onset, metabolic, n (%)	39 (37.1)	17 (33.3)	0.64
RRT during the ICU stay, n (%)	**39 (37.1)**	9 (17.6)	**0.01**

CCI: Charlson comorbidity index; CRP: C-reactive protein; DM: diabetes mellitus; ED: emergency department; ICU: intensive care unit; IQR: interquartile range; IV: intravenous; MDRAB: multidrug-resistant *Acinetobacter baumannii*; MODS: multiple organ dysfunction syndrome; SAPS II: Simplified Acute Physiology Score II; SOFA: Sequential Organ Failure Assessment; RBC: red blood cells; RRT: renal replacement therapy; VAP: ventilator-associated pneumonia; WBC: white blood cells. * polybacterial VAP only due to Gram-negative pathogens; ** Oncology: cancer of a solid organ; *** Obesity: body mass index over 30 kg/m^2^.

**Table 2:** The 30-day mortality of mono- and polybacterial cases of VAP due to MDRAB.

Variable	30-Day Mortality		
	VAP Origin		*p* Value
	Monobacterial, n/Total (%) **	Polybacterial *, n/Total (%) **	
All sample	**60/105 (57.1)**	19/51 (37.3)	**0.02**
Severity on VAP diagnosis			
▪ SOFA < 8	**28/64 (43.8)**	9/35 (25.7)	**0.08**
▪ SOFA > 7	32/41 (78.0)	10/16 (62.5)	0.23
Appropriateness of antibacterial treatment			
▪ Appropriate	**35/69 (50.7)**	8/34 (23.5)	**<0.01**
▪ Inappropriate	25/36 (69.4)	11/17 (64.7)	0.73
Appropriate treatment and severity on VAP diagnosis			
▪ SOFA < 8	18/43 (41.9)	4/21 (19.0)	0.07
▪ SOFA > 7	**17/26 (65.4)**	4/13 (10.3)	**0.04**
Time of appropriate antibacterial treatment			
▪ Early	26/47 (55.3)	6/18 (33.3)	0.11
▪ Late	34/58 (58.6)	13/33 (39.4)	0.08
Antibacterial resistance profile of *A. baumannii* strains			
▪ MDR	9/16 (56.3)	3/7 (42.9)	0.68
▪ XDR	**51/89 (57.3)**	16/44 (36.4)	**0.02**

ICU: intensive care unit; MDR: multidrug-resistant; SOFA: Sequential Organ Failure Assessment; XDR: extensively drug resistant; VAP: ventilator-associated pneumonia. * polybacterial VAP only due to Gram-negative pathogens; ** n: number of deceased patients in the subgroup/total: total of the respective subgroup.

## Data Availability

The data presented in this study are available on request from the corresponding author.
